# Tuberculosis Examinations in the Army

**Published:** 1918-11

**Authors:** 


***Tuberculosis Examinations in the Army.*** By F. B. TRUDEAU (Saranac Lake, N. Y.), New Haven, Conn. *Journal A. M. A.*, Sept. 7th, 1918.

The author reports his experience in examinations for tuberculosis in training camps at Plattsburg and Camp Devens. He states the lessons that have been taught by experience in this work, namely, the grouping of cases in the history taking, as well as in the physical examination itself. He holds that a tuberculosis examining board should occupy only six hours of their time each day in the work, and the number of pulmonary examinations for each medical officer should be at the least seventy-five, but should not exceed 100. The work is so intensive that it is tiring on both mind and body; therefore, the above time limit is suggested. The routine taking of histories is not of much value, but the one most important procedure in the pulmonary examination is auscultation for râles after cough, performed preferably and if possible at the end of respiration. The disability board to which rejections are referred should consist of three members, the president of the examining board, and two others chosen by him from the members of the board. It should have the final disposition of all lung cases referred to it. The average incidence of tuberculosis in the cases examined was 0.5 per cent., slightly lower than in the reserve officers, slightly higher in the National Army, and just this average in the Regular Army.

